# Face negotiation in graduate school: the decision to conceal or reveal depression among life sciences Ph.D. students in the United States

**DOI:** 10.1186/s40594-023-00426-7

**Published:** 2023-05-17

**Authors:** Nicholas J. Wiesenthal, Logan E. Gin, Katelyn M. Cooper

**Affiliations:** 1 Research for Inclusive STEM Education Center, School of Life Sciences, Arizona State University, P.O. Box 874501, Tempe, AZ 85287-4501 USA; 2 Sheridan Center for Teaching and Learning, Brown University, P.O. Box 1912, Providence, Rhode Island 02912 USA

**Keywords:** Mental health, Depression, Graduate school, Graduate research, Graduate teaching, Concealable stigmatized identities

## Abstract

**Background:**

Depression is one of the top mental health concerns among biology graduate students and has contributed to the “graduate student mental health crisis” declared in 2018. Several prominent science outlets have called for interventions to improve graduate student mental health, yet it is unclear to what extent graduate students with depression discuss their mental health with others in their Ph.D. programs. While sharing one’s depression may be an integral step to seeking mental health support during graduate school, depression is considered to be a concealable stigmatized identity (CSI) and revealing one’s depression could result in loss of status or discrimination. As such, face negotiation theory, which describes a set of communicative behaviors that individuals use to regulate their social dignity, may help identify what factors influence graduate students’ decisions about whether to reveal their depression in graduate school. In this study, we interviewed 50 Ph.D. students with depression enrolled across 28 life sciences graduate programs across the United States. We examined (1) to what extent graduate students revealed their depression to faculty advisors, graduate students, and undergraduates in their research lab, (2) the reasons why they revealed or concealed their depression, and (3) the consequences and benefits they perceive are associated with revealing depression. We used a hybrid approach of deductive and inductive coding to analyze our data.

**Results:**

More than half (58%) of Ph.D. students revealed their depression to at least one faculty advisor, while 74% revealed to at least one graduate student. However, only 37% of graduate students revealed their depression to at least one undergraduate researcher. Graduate students’ decisions to reveal their depression to their peers were driven by positive mutual relationships, while their decisions to reveal to faculty were often based on maintaining dignity by performing preventative or corrective facework. Conversely, graduates performed supportive facework when interacting with undergraduate researchers by revealing their depression as a way to destigmatize struggling with mental health.

**Conclusions:**

Life sciences graduate students most commonly revealed their depression to other graduate students, and over half reported discussing depression with their faculty advisor. However, graduate students were reluctant to share their depression with undergraduate researchers. Power dynamics between graduate students and their advisors, their peers, and their undergraduate mentees influenced the reasons they chose to reveal or conceal their depression in each situation. This study provides insights into how to create more inclusive life science graduate programs where students can feel more comfortable discussing their mental health.

**Supplementary Information:**

The online version contains supplementary material available at 10.1186/s40594-023-00426-7.

## Introduction

### Depression in graduate researchers

Depression is one of the most frequently reported mental health concerns among graduate students (American College Health Association, 2021; Evans et al., 2018; Forrester, 2021), and recent studies indicate that depression may be more common among graduate students compared to their peers in the general public (Bolotnyy et al., 2020; Evans et al., 2018). Depression is described by the American Psychiatric Association as “a common and serious mental illness that negatively affects how one feels, thinks, and acts” (American Psychiatric Association, 2020).

Depression is thought to be particularly prominent among both undergraduate and graduate science students (Busch et al., 2022a; Evans et al., 2018). The chilly and sometimes unwelcoming nature of science courses and uniquely competitive science environments can contribute to student feelings of isolation and sadness (Araghi et al., 2023; Cooper et al., 2020a; Gin et al., 2021; Seymour et al., 2019). Depression among graduate students in particular can also be exacerbated by financial stress (Charles et al., 2021; Hish et al., 2019; Jones-White et al., 2021), lack of a work–life balance (Evans et al., 2018; Liu et al., 2019), and poor advisor–advisee relationships (Charles et al., 2021; Evans et al., 2018; Hish et al., 2019; Liu et al., 2019; Peluso et al., 2011). Conducting graduate research has been identified as a specific aspect of science graduate programs that can worsen depressive symptoms (Gin et al., 2021); science Ph.D. students report that encountering failure and being tasked with carrying out unstructured research projects can be particularly challenging. In addition, aspects of graduate teaching, including negative reinforcement from undergraduates in the form of poor teaching evaluations, and feeling unprepared to teach, can also exacerbate depressive symptoms among science graduate students (Gin et al., 2021). Furthermore, recent research suggests that graduate students with depression are more likely consider leaving their science graduate programs compared to their counterparts who do not have depression (Busch et al., unpublished data).

Given the substantial negative effects that depression can have on science graduate students’ social lives (Gin et al., 2021; Steger & Kashdan, 2009), professional lives (Hish et al., 2019; Peluso et al., 2011), and overall quality of life (Eisenberg et al., 2007; Evans et al., 2018), the scientific community is increasingly recognizing the importance of improving graduate student mental health (“The Mental Health of PhD Researchers Demands Urgent Attention”, 2019; National Academies of Sciences, Engineering, & Medicine, 2021; Pain, 2018; Puri, 2019; Woolston, 2020). While efforts have been made to increase access to student mental health resources (Kodish et al., 2021; Lipson et al., 2019), students would likely also benefit from graduate programs and research advisors fostering graduate school environments that promote mental health (Gin et al., 2021; Langin, 2021).

### Depression as a concealable stigmatized identity

Helping science graduate students cope with depression and alleviating aspects of graduate school that exacerbate student depression may be uniquely difficult due to the concealable nature of this identity. Specifically, depression is considered a concealable stigmatized identity (CSI), meaning that it can often concealed and is associated with negative stereotypes that can result in discrimination and loss of status in society (Link & Phelan, 2001; Quinn & Earnshaw, 2011). Concealable stigmatized identities also include other mental illnesses, LGBTQ + identities, and chronic health conditions (A. H. Crisp et al., 2000; Earnshaw & Quinn, 2012; Link et al., 2001; Meyer, 2003). Individuals with CSIs are faced with the unique decision to reveal or conceal their identity in social situations. Individuals who reveal CSIs report experiencing judgement, stigmatization, or prejudiced treatment (Chaudoir & Fisher, 2010; Flett, 2012; Pachankis, 2007; Ragins et al., 2007), and this has been shown to be true for individuals with depression in the workplace (Evans-Lacko et al., 2012; Krupa et al., 2009; Ridge et al., 2019). While CSIs are commonly concealed in effort avoid such discrimination, research has shown that concealing the identity can also lead to psychological distress (Quinn & Chaudoir, 2009; Quinn et al., 2014). However, when one is able to reveal their CSI to an accepting confidant, the psychological distress can be alleviated and the individual may experience increased emotional support (Black & Miles, 2002; Cooper et al., 2020b; Corrigan & Matthews, 2003).

### Choosing to conceal and reveal depression in science graduate programs

Studies of how individuals navigate their CSIs primarily focus on work environments (Flett, 2012; Follmer & Jones, 2018; Webster et al., 2018; Yoder & Mattheis, 2016), academic environments (Cooper et al., 2020b; Busch et al., 2023; Cooper et al., 2019; England, 2016; Varjas et al., 2016; Yerbury & Yerbury, 2021), or personal environments (Brooks et al., 2018; Kaushansky et al., 2017; Pahwa et al., 2017). However, science graduate programs provide a unique context to explore the revealing of CSIs, since they encompass a unique blend of professional duties, academic expectations, and personal relationships. Notably, graduate students operate within an array of power structures; they interact with superiors (e.g., primary investigators), peers (e.g., fellow graduate students), and subordinates (e.g., undergraduate researchers). Depression can negatively affect graduate students’ performance in research, and graduate students with depression perceive that a change in their performance is sometimes noticed by advisors, fellow graduate students, and undergraduates working with the graduate student (Gin et al., 2021). However, it is unknown to what extent graduate students choose to discuss their depression with individuals with whom they do research. We hypothesize that the power dynamics within each of these relationships may influence how science graduate students navigate their depression.

### Theoretical framework: face negotiation theory

Previously, our research group has drawn from the Disclosure Decision Model (DDM) (Omarzu, 2000) and the Disclosure Process Model (DPM) (Chaudoir & Fisher, 2010) to understand individuals’ decisions to reveal their depression in academic environments (Cooper et al., 2020b). Briefly, the DDM describes why individuals decide to self-disclose personal information in various types of situations (Omarzu, 2000); it suggests that individuals need to possess one of the five possible goals related to disclosing their CSI: (1) approval, (2) intimacy, (3) relief, (4) identity, or (5) control. The DPM highlights when and why self-disclosure can be beneficial to the individual. Specifically, disclosing one’s CSI can result in three scenarios: (1) it can alleviate inhibition or psychological distress, (2) it can promote social support, and (3) it can change the social information between the discloser and their confidant (Chaudoir & Fisher, 2010). While both models provide important insight about when and why graduate students choose to disclose their depression, we argue that understanding the decision to *conceal* depression requires the consideration of additional theories.

We draw from the concept of facework and face negotiation theory to further our understanding of graduate students’ decisions to reveal or conceal their depression in the context of research environments. The term “face” refers to a favorable sense of social self-worth (Ting-Toomey, 1982; Ting-toomey & Kurogi, 1998). Face negotiation theory describes the process of facework, defined as “a set of communicative behaviors that individuals use to regulate their social dignity or that of others”(Ting-toomey & Kurogi, 1998, pg. 188). Traditionally, face-management has been referenced when analyzing common situations in business including negotiation and conflict management. However, we propose that face-management is applicable to graduate school, as Ph.D. students interact with faculty advisors, fellow graduate students, and undergraduate mentees and engage in exchanges, negotiations, and, at times, conflict management (Gin et al., 2021).

One’s culture(s) is assumed to play a key role in face-management (Ting-toomey & Kurogi, 1998). Theorists distinguish between small power distance cultures, defined as cultures where equal power distributions are valued, and large power distance cultures, defined as cultures where hierarchical roles are valued (Hofstede, 1991). The U.S. is thought to have a medium power-distance culture where subordinates’ dependency needs are neither too high nor too low (Hofstede, 1991). However, academia is notorious for having a large power distance culture, particularly between tenure-track/tenured faculty and the staff and students at a college or university (Tormey, 2021; Xu & Ran, 2021). The power dynamics between two individuals often dictate the types of facework that individuals engage in (Ting-Toomey & Kurogi, 1998). Assumptions about different cultures have resulted in the description of cultural-specific facework, such as Chinese facework (Bond, 1991), Mexican facework (Garcia, 1996), and Japanese facework (Morisaki & Gudykunst, 1994). We argue that it is worth considering the culture of academia when discussing facework in the context of graduate research, particularly as it relates to whether one chooses to reveal or conceal a CSI, such as depression.

Ting-Toomey and Cole (1990) describe three styles of facework, or communication behaviors that people use to maintain social dignity. **Preventative facework (face saving)** refers to a strategy to prevent conflict by preparing others about potential conflicts and proactively avoiding them (Ting-toomey & Kurogi, 1998, p. 191). **Supportive facework** refers to a strategy that assumes mutual respect, security, and value among those who are interacting, and it often reflects the way that one would want to be treated by others (Ting-toomey & Kurogi, 1998, p. 192). Finally, **corrective facework (face restoration)** encompasses strategies deployed to save face, or repair one’s reputation, often by offering explanations or apologizing for mistakes (Ting-toomey & Kurogi, 1998, p. 191).

### The potential benefits of science graduate students revealing depression

We posit that understanding what influences graduate students’ decisions to reveal and conceal their depression will inform how to create a more inclusive scientific community. If science graduate students can be more open about their depression, particularly if they wish to be, this will likely result in more institutional efforts to bolster graduate mental health. Given that depression disproportionately impacts individuals who are underrepresented and underserved in science, including women (American College Health Association, 2018; Evans et al., 2018), first-generation college students (Jenkins et al., 2013), individuals from low socioeconomic backgrounds (Eisenberg et al., 2007), members of the LGBTQ + community (Eisenberg et al., 2007; Evans et al., 2018) and individuals with disabilities (Turner & Noh, 1988), such efforts are likely integral to creating a more diverse and inclusive scientific community (Intemann, 2009). In addition, graduate students have the potential to serve as role models for undergraduate students who have depression. Research has shown that science students are unlikely to know of scientists with depression (Cooper et al., 2020b; Gin et al., 2021), despite depression being relatively common among professionals in the sciences (Busch et al., unpublished data). Yet, undergraduates with and without depression have identified an array of ways that knowing a scientist with depression could be helpful to them; undergraduates report that knowing a scientist with depression causes them to feel as though they belong more to the scientific community and to perceive the scientific community as more inclusive (Cooper et al., 2020b, Mohammed et al., unpublished data). Therefore, while we recognize that choosing to reveal one’s depression is a deeply personal decision and will not always be a positive experience (Chaudoir & Quinn, 2010), we aim to identify what factors influence science graduate students’ decisions to reveal and conceal their depression in hopes of illuminating ways to promote mental health among scientists.

## Current study

In this study, we examine Ph.D. students’ decisions to reveal or conceal their depression in the context of research, with the intent to identify differences in the motivation of Ph.D. students’ choices to reveal or conceal their identity to faculty advisors, fellow graduate students, and undergraduate researchers. Our research questions are:

- To what extent and why do Ph.D. students reveal or conceal their depression to faculty advisors, fellow graduate students, and undergraduate researchers in the context of their research experiences?
- What do Ph.D. students perceive to be the benefits and consequences of revealing their depression to faculty advisors, graduate students, and undergraduate researchers in the context of their research experiences, and do these perceptions differ between students who have revealed their depression and those who have chosen to conceal it?

## Methods

### Student interviews

This study was conducted with an approved Arizona State University Institutional Review Board protocol #00011040.

In Fall 2019, we recruited life sciences Ph.D. students to participate in a survey study about their experiences in graduate school. We sent an email to every program administrator of U.S. life science graduate programs recorded in U.S. News and World Report (U.S. News & World Report, 2019). Out of the 259 graduate programs that were contacted for this study, 75 (29%) sent our survey to students registered in their respective Ph.D. program. Of the 840 graduate students who completed the survey, 459 (55%) students self-identified as having depression and 327 (71%) of those students expressed interest in participating in follow-up interview. We used this list of students to recruit for the current study.

We sent a recruitment email out to the 327 Ph.D. students with depression in the summer of 2020 inviting them to be interviewed about their experiences with depression while enrolled in a life sciences Ph.D. program. Because mental healthcare is often made inaccessible for Black and Latin* individuals, as well those from low socioeconomic backgrounds (Howell & McFeeters, 2008; Kataoka et al., 2002; Santiago et al., 2013), we did not require students to be clinically diagnosed with depression to participate in this interview study. A total of 50 Ph.D. students (15% of those contacted) from 28 life sciences Ph.D. programs participated in an interview.

We developed an interview script to answer our research questions based on a previous interview script used to elicit information about undergraduates’ decisions to reveal or conceal their depression in undergraduate research experiences (Cooper et al., 2020b). We adapted this script to probe whether Ph.D. students have revealed their depression to at least one faculty advisor, one graduate student or postdoc, and one undergraduate researcher with whom they work with regularly in a research environment. For example, instead of asking undergraduates whether they concealed or revealed their depression to their graduate mentors, we asked graduate students whether they revealed or concealed their depression to their undergraduate mentees. Specifically, we explored students’ reasoning for either choosing to reveal or conceal their depression in each situation; however, if graduate students did not report working regularly with a faculty research mentor, graduate students/postdocs, or an undergraduate, they were not asked the related questions about revealing/concealing their depression. All participants regularly worked with a faculty mentor, and all but three students regularly worked with at least one graduate student or postdoc; the majority discussed fellow graduate students in their interviews. The three who did not had joined new faculty member labs where they were the only graduate student researcher. Thirty-eight of the 50 graduate students reported working regularly with an undergraduate researcher. We asked Ph.D. students who concealed their depression about the potential benefits and consequences they perceived they would encounter if they did reveal their depression to others in their lab. Relatedly, we asked students who chose to reveal their depression to others in the lab what benefits and consequences they actually experienced after revealing.

All interviews were conducted during the COVID-19 pandemic in summer of 2020, and we hypothesized that students’ depression was likely exacerbated because of these circumstances (Chirikov et al., 2020). As such, we asked students to consider their experience in research prior to the pandemic when answering the interview questions. To ensure that the interview questions were understandable and were in no way offensive to students with depression, we conducted think-aloud interviews with four graduate students who self-identified as having depression (Trenor et al., 2011). After each think-aloud interview, the researchers revised the interview script until all interview questions appeared to be interpreted by the interviewee as intended. The final copy of the interview script can be found in the Additional file 1.

The interviews were conducted using Zoom by one of two researchers (L.E.G. or K.M.C.) and averaged 45 min. All participants were sent a survey after the interview to collect additional information regarding their depression and demographics. The finalized survey sent out to the participants can be found in the Additional file 1. Students were given a gift card in exchange for the time they spent participating in the interview. To protect the participants’ identities, all interviews were deidentified before being transcribed, and all interview participants have been given a pseudonym.

### Interview analysis

We used a hybrid approach of deductive and inductive coding to answer our research questions (Fereday & Muir-Cochrane, 2006). We used deductive coding to identify whether Ph.D. students reported revealing their depression to faculty advisors, fellow graduate students, and undergraduate researchers. We used inductive coding methods to allow themes to emerge from students’ responses (Saldaña, 2015). Specifically, we examined students’ responses for reasons why they chose to reveal or conceal their depression and the benefits and consequences they associated with revealing in three separate situations: to faculty advisors, to graduate students, and to undergraduate researchers. Three researchers (L.E.G., K.M.C., and N.J.W.) were assigned a randomly selected set of 12 interviews to review for common themes and individually annotated each interview. The researchers then came together to discuss their findings and create an initial codebook of emergent themes. Each of the three researchers reviewed a separate randomly selected set of five interviews to validate the initial themes outlined in the codebook and to see if any additional themes emerged. The researchers then came back together and used constant comparison methods to further validate all themes by comparing quotes from the interviews they reviewed to their respective themes (Glesne & Peshkin, 2016). If quotes were too different from the themes outlined in the codebook, a new theme was created (Glesne & Peshkin, 2016). A copy of the final codebook can be found in the Additional file 1. The final codebook was used by two researchers (L.E.G. and N.J.W.) to individually code a newly randomly selected set of five interviews (10% of all interviews), and the researchers’ interrater score was rated at an acceptable level (k = 0.94, (Landis & Koch, 1977). The remaining 45 interviews were then coded by one author (N.J.W.). Quotes from the interviews were used to demonstrate themes that were reported by at least 10% of the participants. However, it is important to note that because the interview questions were open-ended, it is likely that the percentage of students who identify with each theme is underreported. Some student quotes were slightly edited for grammar or clarity.

We examined whether there were trends in revealing depression with regard to whether a student’s depression was formally diagnosed, the self-reported severity of their depression, and the number of years a student had been in their graduate program. For each of these inquiries, we divided the number of times a particular group revealed their depression by the number of possible opportunities to reveal and qualitatively compared the results.

### Author positionality

Some of the authors identify with having depression and some do not. The researchers’ personal experiences with depression informed the coding rubric; the researcher(s) perceived that having depression made it easier to identify themes that were emerging, while not having depression helped counteract any potential biases (Chenail, 2011). At the time of the interviews, one of the authors had completed a Ph.D. program and was serving as a research advisor (K.M.C.), and two authors were in enrolled in a graduate program (L.E.G. and N.J.W.).

## Results

### Participant demographics

Of the 50 life sciences Ph.D. students who completed interviews, students most commonly identified as being a woman (58%), white (74%), and as having been a continuing generation college student (78%). Students primarily identified as either being in the second (26%) or third (24%) year of their Ph.D. program. Participants’ depression during their Ph.D. program was primarily moderate (50%) or severe (28%), and nearly 75% reported being treated for depression. All student demographic data are summarized in Table 1.

**Table 1:** Demographics of the 50 interview participants

Student-level demographics	*n* = 50 *n* (%)	Research/teaching demographics	*n* = 50 *n* (%)	Depression demographics	*n* = 50 *n* (%)
**Gender**		**Program year**		**Severity during graduate school**	
Woman	29 (58%)	1st year	4 (8%)	Mild	7 (14%)
Man	17 (34%)	2nd year	13 (26%)	Moderate	25 (50%)
Non-binary/Gender fluid	4 (8%)	3rd year	12 (24%)	Severe	14 (28%)
		4th year	5 (10%)	Extremely severe	4 (8%)
**Race/ethnicity**		5th year	7 (14%)	**Formally diagnosed with depression**	
Asian/Pacific Islander	4 (8%)	6th year or more	6 (12%)	Yes	41 (82%)
Black/African American	1 (2%)	Recently graduated	3 (6%)	No	7 (14%)
Hispanic/Latin’	4 (8%)			Decline to state	2 (4%)
White/Caucasian	37 (74%)				
> 1 race/ethnicity	3 (6%)				
Decline to state	1 (2%)				
**College generation statu**s					
First-generation	11 (22%)				
Continuing generation	39 (78%)				

### Graduate students most commonly revealed their depression to fellow graduate students and infrequently revealed their depression to undergraduate researchers

Graduate students frequently reported that they had revealed their depression to at least one other graduate student (74%), while more than half had revealed their depression to their faculty advisor (58%). Of the 38 graduate students who reported that they regularly worked with at least one undergraduate researcher, only 37% reported that they had revealed their depression to an undergraduate. We examined whether there were trends in revealing depression based on whether a student’s depression was formally diagnosed, the self-reported severity of the student’s depression, and the number of years a student had been in their graduate program. There were very few differences in the extent to which students revealed depression among students who had and had not been formally diagnosed with depression and no clear trend based on year in the program. The most obvious trend related to severity of depression: as depression severity increased, students more commonly revealed their depression. A table that lists each study participant, whether they have been formally diagnosed with depression, their self-reported severity of depression during graduate school, the number of years they had been in their graduate program at the time of the interview, and whether they reported revealing depression to a faculty mentor, a graduate student, and an undergraduate researcher is included the Additional file 1 along with the results of each query. Below we report graduate students’ reasoning for revealing or concealing their depression to faculty advisors, graduate students, and undergraduate researchers. We also summarize the benefits and consequences students experienced or predicted they would experience as a result of revealing depression in each situation.

### Revealing and concealing depression to faculty advisors

***Ph.D. students primarily revealed their depression to faculty advisors because their depression was affecting their research or they worried their depression would eventually affect their research. ***The 29 graduate students who revealed their depression to faculty advisors commonly reported four reasons why **(**Table 2**)**. Nearly half of the students who revealed their depression (48%) described engaging in preventative facework; they perceived depression would or already was affecting their attendance in the lab or productivity in research and pre-emptively wanted to let their faculty advisor know to prevent future judgement or conflict. Conversely, 41% of graduate students reported engaging in corrective facework after depression interfered with their mood or behavior in research and it was noticed by others; they revealed their depression as a means of explaining the noted behavioral or mood change. For example, some students described that their faculty advisor inquired about their mood or behavior when they were particularly sad or upset, or when they were absent from the lab. In addition, students described situations where they experienced extreme sadness or hopelessness while in the presence of their faculty advisor, such as when crying in a one-on-one meeting. In addition, graduates reported revealing their depression to faculty advisors because they perceived they would not be judged, either because their faculty advisor had demonstrated that they were an understanding individual (22%) or because they had developed a close personal relationship with the graduate student (17%).

**Table 2:** Reasons why Ph.D. students revealed their depression to their faculty advisor

Theme	Description	Percent (*N* = 29)^a^	Example student quote 1	Example student quote 2
Preventative reveal	Student perceived that depression would impact or was impacting their mood or performance in research and revealed depression preemptively to avoid judgement or conflict.	48%	Student 6: “I had to come clean to [my faculty advisor] a couple of times. If I was coming into the lab less, I would say, ‘Hey advisor, I am struggling with depression right now. I am taking proactive steps to do this, this, and this about it.’”	Student 34: “Hiding [my depression] doesn't help because if I don't mention it then [my faculty advisor] is not going to know why my productivity comes and goes in waves.”
Corrective reveal	Student reports that depression negatively affected their mood or performance in research, which was noticed by others. The student revealed their depression in an attempt to explain their mood or behavior.	41%	Student 12: “We had a lab meeting that was just a rough day for me. [My faculty advisor] emailed me afterwards and said, ‘Hey. You didn't look good today. Are you okay? What's up?’ I decided to just be forthright with her and tell her what was going on [with my depression].”	Student 5: “It was actually after a committee meeting [when I revealed my depression]. I was talking about career stuff and [my faculty advisor] pulled me aside the next day and was just like, ‘Hey, you seem really depressed.’ And I was like, ‘Well, I am.’ And it was a really hard conversation.”
No judgment—understanding	Student perceived that they would not be judged if they revealed because their advisor’s past actions indicated that the advisor is understanding, caring, or has a positive perception of mental health.	22%	Student 3: “My advisor laid out some very clear objectives for herself as our advisor. Part of it wasn't directly stating like, ‘Hey, as your advisor, I want to help you with your mental health,’ but it was, ‘As your advisor, I want to help you as a person.’”	Student 8: “[My faculty advisor] has always been very upfront [about mental health], even before she knew [about my depression]. She was like ‘I always want to be a support system for my graduate students, so if you’re struggling with anything talk to me and I will help you handle that because that’s my job as your advisor.”
No judgment—personal relationship	Student perceived that they would not be judged for revealing because they had developed a close personal relationship with their faculty advisor.	17%	Student 13: “My priority was making sure that [my faculty advisor] was someone that I could become friends with. So, from the get-go I knew that if something happened [with my depression] I could talk to him about it.”	Student 9: “[My faculty advisor] made me feel very comfortable in telling her about things I'm going through [with my depression]. Our friendship really contributed to the fact that I was comfortable with her and that I could reveal to her what I was feeling without fearing that she would judge me.”

^a^Percentages add to more than 100 because students often described more than one reason for revealing their depression to their faculty advisor

***Ph.D. students primarily concealed their depression from faculty because they worried about being perceived negatively or because they are not personally comfortable with their depression.*** Five common themes emerged as to why graduate students concealed their CSI from their faculty advisors **(**Table 3**)**. Students most commonly feared that their faculty advisor would perceive or treat them differently or negatively (57%). Students used their faculty advisor’s past behaviors toward mental health issues or other marginalized identities to gauge how the advisor might respond to knowing that they had depression. For example, students described noting when their peers brought up mental health and their advisor perceived they were making an excuse for a change in productivity. Furthermore, students also concealed their depression if they perceived a cultural or age difference between themselves and their faculty advisor (19%). For example, students who had advisors who they considered much older or who they perceived had an easy time in graduate school were reluctant to reveal their depression because they assumed their advisor would not understand. Concealing to avoid being judged or concealing to avoid conflicting opinions are both reflective of preventative facework, since students are concealing to prevent negative interactions and stereotyping. Some students perceived that revealing their depression to their faculty advisor was unnecessary (29%) because it did not affect their performance in graduate school, and some (14%) felt that revealing their depression to their faculty advisor would be inappropriate. Participants who described that revealing their depression would be inappropriate often discussed a lab culture that encouraged students to separate their personal lives and emotions from their work. Finally, 38% of students highlighted that they are personally uncomfortable with their depression and, therefore, do not reveal it to anyone.

**Table 3:** Reasons why Ph.D. students concealed their depression from their faculty advisor

Theme	Description	Percent (*N* = 21)^a^	Example student quote 1	Example student quote 2
May be perceived or treated differently or in a negative way	Student describes they were afraid they would be perceived or treated negatively if their advisor knew of their depression. Students often report using their advisor’s prior behaviors to predict how they would react.	57%	Student 2: “I guess it's really just that idea of like, I don't want to be treated differently [because of my depression]. I don't want to be handled with kid gloves. I want to be able to say when I get through grad school that I got through it just like everybody else.”	Student 18: “I guess I really don't want [my faculty advisor] to see me as only someone [who has depression], or really see me through a lens of ‘oh my student is depressed.’ I don't want her to think that I am making any excuses.”
Is uncomfortable with depression	Student describes that they are personally uncomfortable sharing their depression with anyone.	38%	Student 44: “I think it was mostly that I didn't feel comfortable [revealing my depression] because I sometimes still don't feel comfortable sharing it with other people.”	Student 50: “I have always not felt super comfortable talking about [my depression] with people in general, and especially people that I work for.”
Perceives revealing to be unnecessary	Student did not think it was necessary to share their depression, often because they did not perceive it affected their work.	29%	Student 17: “I decided not to [reveal my depression] explicitly because I didn't think it was necessary. I think if something changed and I felt like it was affecting me a lot more and it would be helpful for [my faculty advisor] to know, then I would tell [my advisor].”	Student 27: “[My depression] has never been something that's really affected my ability to do things. So, it's never been something that was necessary to bring up.”
Perceives an identity, cultural, or age difference between themselves and their advisor	Student does not reveal because they worry that the faculty advisor’s identity, culture, or age would prevent them from understanding what the student is trying to explain about depression.	19%	Student 14: “[My faculty advisor] is in her late fifties, so she comes from a different generation. Even her response to different things makes me feel like she wouldn’t be able to respond [to my depression].”	Student 43: “So [my faculty advisor] will constantly remind me and other students that grad school is supposed to be the best time of your life. He just doesn't seem to understand why and how grad school would be very hard [on someone’s depression]. Because I guess for him, it was a very enjoyable stress-free experience.”
Perceives revealing to be inappropriate	Student perceives that it is inappropriate to reveal depression in a lab environment, often because of the assumption that emotion is not welcome in science.	14%	Student 39: “It's just [my faculty advisor’s] personality where emotional or personal problems don't necessarily have to interfere with your work.”	Student 14: "I came from a very competitive [graduate] program, and I think being kind to [your mental health] was not part of the culture. I remember telling someone I worked with [in my program] that I struggled with [depression] and they said, 'Oh, really? When we were young, I guess these things happened. And we just used to sort of get over it.' And I think that has stayed with me."

^a^Percentages add to more than 100 because students often described more than one reason for concealing their depression to their faculty advisor

***Ph.D. students perceived that the primary benefits of revealing their depression to their faculty advisor are increased flexibility and support.*** Among the 29 students who revealed their depression, students commonly highlighted that their faculty advisor became more flexible or understanding after learning of their depression (48%). For example, students explained that their faculty advisors were sometimes more understanding of their outward emotions (e.g., sadness) or behaviors (e.g., missing a deadline) related to their depression. However, only 19% of the 21 students who had concealed their depression anticipated that revealing it would result in such flexibility. Both students who revealed their depression and those who concealed it commonly agreed that being open about their depression resulted (48%), or could result (43%), in their faculty advisor being more supportive of them. For example, students who had revealed their depression described their faculty advisor being more likely to check in on them or provide opportunities to talk about how their work was affecting their depression or vice versa. Finally, students felt that by revealing their depression they were able to be more honest with their faculty advisor if they could not fulfill a research-related obligation or meet a deadline (10%), yet no student who concealed their depression mentioned this theme.

***Ph.D. students perceived that the primary consequence of revealing one’s depression is being judged by their faculty advisor.*** Graduate students who concealed their depression from their faculty advisor identified more consequences associated with revealing than those who actually revealed their depression. Students who revealed their depression sometimes highlighted that they felt judged by their faculty advisor after sharing their depression (21%), which was a common fear of those who concealed (24%). Students who concealed their depression feared that revealing it could result in being prevented from carrying out their usual responsibilities as a graduate student (14%), but this was not reported as a consequence by students who revealed.

### Revealing and concealing depression to fellow graduate students

***Ph.D. students primarily revealed their depression to fellow graduate students because they had established a personal relationship.*** Graduate students commonly reported six factors that motivated them to reveal their depression to their peers (Table 4). Most commonly students discussed that they were willing to reveal because they would not be judged, owing to a personal relationship (49%), a mutual struggle with mental health (43%), or shared negative experiences in graduate school (20%). Students also reported that they revealed their depression to other graduate students in an act of supportive facework: to serve as a role model and destigmatize mental health, often in hopes that their peers might feel comfortable speaking about their own mental health (20%). Notably, graduate students were less likely to engage in preventative facework by revealing their depression to preemptively avoid conflicts or concerns resulting from changes in their mood or behavior in lab (17%), or corrective facework by revealing depression to offer an explanation after others commented on a change in their mood or behavior (11%).

**Table 4:** Reasons why Ph.D. students revealed their depression to other graduate students

Theme	Description	Percent (*N* = 35)^a^	Example student quote 1	Example student quote 2
No judgement—personal relationship	Student reports that they did not think they would be judged for revealing because they had developed a close personal relationship with a fellow graduate student.	49%	Student 3: “[The other graduate students and I] are just a supportive group. (…) From the beginning, my advisor set an environment where she wants it to be a family. Family can mean different things for different people, but at least in our family unit, we try to be there for each other.”	Student 23: “[The other graduates and I] are a family in my lab. We have some kind of relationship that's in-between a sibling and coworker. And I socialize with them a lot on the weekends and on weeknights, and we eat lunch together every day, and we talk all the time.”
No judgement—other person struggles with mental health	Student reports that they did not think they would be judged for revealing because the other graduate student also struggles with mental health.	43%	Student 5: “I think [another graduate student] mentioned that he was vaguely struggling [with depression] and I was like, ‘No way. Me too.’ So, we sat down and had lunch together and it all really came out. We were just like, ‘Dude, we're going through the same things.”’	Student 8: “The fact that [another graduate student] revealed to me that he was experiencing [depressive symptoms] when he was getting his graduate degree made me feel like he could understand my experience.”
No judgement – shared (often negative) experiences in graduate school	Student reports that they did not think they would be judged for revealing because they shared similar experiences, often negative ones, with another student during graduate school.	20%	Student 4: “There's such a sense of camaraderie in which [the other graduate students and I] are all experiencing this together, and so it was really easy [to reveal my depression]. The other graduate students at my institution talk about really personal issues all the time and are very supportive as a group in general.”	Student 17: First you're talking about things that you and the other student both worry about, and then those conversations become deeper, and more than just like, ‘I'm worried about how this experiment will go. I'm disappointed about this.’ Over time it becomes more [about depressive symptoms] like, ‘I don't know if I can do this. There's something that might be wrong with me.’”
Supportive reveal	Student wanted to reveal their depression to normalize struggling with mental health.	20%	Student 21: “I've just felt like I'm instilling the groundwork and [the other graduate students and I] should be allowed to talk about anything because we're suffering through this together. (…) So I was trying to make it comfortable for everyone to talk about [their mental health].”	Student 23: “It was really important to me that I share my experience because I think that mental illness is so isolating, it's really important to be a role model so that it can be accepted and let [my peers] know that other people around you are suffering with [depression].”
Preventative reveal	Student perceived that depression would impact or was impacting their mood or performance in research and revealed depression preemptively to avoid judgement or conflict. Once others notice or comment on one’s behavior related to depression the reveal becomes corrective instead of preventative.	17%	Student 26: " I was tired of being afraid that [the other graduate students] would figure out that I was lying [about why I was absent], or people would think that I was sketchy or unreliable on purpose, and so over the years, I've just decided that being honest about [my depression] is less stressful for me, and easier for me."	Student 40: “We collaborated a lot, and I was working with her on writing a paper and it just kind of got to the point where things probably weren't adding up to her. I gave her a general heads up and said, ‘I'm really sorry I'm not making as much progress on this. I've kind of been struggling with some mental health stuff and I'm doing the best I can.’”
Corrective reveal	Student reports that depression negatively affected their mood or performance in research, which was noticed by others. The student revealed their depression in an attempt to explain their mood or behavior.	11%	Student 4: “[The other graduate students] saw me getting worse and worse [with my depression] during my first year of graduate school. It was physically obvious because I would show up in the same clothes that I was wearing yesterday and was clearly very tired and sometimes checked out.”	Student 31: “I was having a really bad depressive episode in the middle of the lab. (…) My heart was going like a million miles an hour and I just started crying my eyes out at my desk after having closed the door. [Another graduate student] walked in five minutes later and saw me obviously in tears.”

^a^Percentages add to more than 100 because students often described more than one reason for revealing their depression to other graduate students

***Ph.D. students were reluctant to reveal their depression to fellow graduate students when they did not have a close personal relationship with them or if they were afraid of being treated differently.*** The 26% of students who concealed their depression from other graduate students highlighted four common reasons why (Table 5). Like with those who revealed their depression, students’ personal connections with others most commonly affected their decision; specifically, graduate students often highlighted that the lack of a personal relationship with other graduate students in their lab prevented them from revealing their depression (58%). Participants also reported that they concealed their depression due to a fear of being judged or perceived negatively by other fellow graduate students within their lab (33%), a form of preventative facework (Brown, 1977; Ting-Toomey & Cole, 1990; Ting-toomey & Kurogi, 1998). Some students highlighted that their depression does not affect their research, as such, they find revealing their depression to other graduate students to be unnecessary (17%), and some generally feel uncomfortable with revealing their depression with others regardless of whom they are (17%).

**Table 5:** Reasons why Ph.D. students concealed their depression from other graduate students

Theme	Description	Percent (*N* = 12)^a^	Example student quote 1	Example student quote 2
Lack of personal relationship	Student describes that they did not reveal their depression because they did not establish a close enough relationship with another graduate student.	58%	Student 13: “[The other graduate students and I] never got that close. I can remember instances where we were developing a closer bond and I would have been more comfortable sharing [my depression], but we just didn't get to that.”	Student 47: “I've never had a close friendship with anyone directly in my lab. We get along fine in day-to-day interactions and stuff like that, but none of the people who have gone through my lab, either students, postdocs, or otherwise, have been close enough friends that [my depression] is something I would talk about with them.”
May be perceived or treated in a negative way	Student describes they were afraid they would be perceived or treated negatively if other graduate students knew of their depression. Students often report using their fellow graduate students’ prior behaviors to predict how they would react.	33%	Student 44: “With [graduate students], I think it is that I don't want them to treat me differently, but I also don't really want them to judge me for [my depression].”	Student 45: “[The other graduate students] might not know how to respond to [my depression] or I guess might treat me a little bit differently based on that as opposed to ADHD, which I feel like there's a lot less stigma about.”
Perceives revealing to be unnecessary	Student did not think it is necessary to share depression, often because they did not perceive it affected their work.	17%	Student 37: “I just don't think [revealing my depression] is relevant. I'm all about talking about mental health and supporting one another, but I felt like I had enough of a support system with my boyfriend and my family that I didn't need to.”	Student 41: “It's just when it comes to [concealing my depression], I know I have a job to do. I just go about my own business.”
Is uncomfortable with depression	Student describes that they are personally uncomfortable sharing their depression with anyone.	17%	Student 22: “It's just not something that I've spoken about with a lot of people. So, I'm just not the greatest at talking about it in general.”	Student 50: “For the graduate students, I just don't like to talk about [my depression], as I wouldn’t with anyone else, so it’s less about that professional barrier, and more of a personal preference.”

^a^Percentages add to more than 100 because students often described more than one reason for concealing their depression to other graduate students

***The most commonly perceived benefit of revealing depression to fellow graduate students is increased support.*** Some of the benefits associated with revealing depression to faculty advisors also applied to revealing depression to fellow graduate students. Both students who concealed (67%) and revealed (80%) their depression commonly highlighted that revealing one’s depression may result in or actually resulted in forming stronger relationships with fellow graduate students and receiving increased support demonstrated by checking in more frequently or helping with research tasks. In addition, students who revealed their depression also described that they could be more honest with other graduate students if they were unable to fulfill their duties as a graduate student because of their depression (14%). However, this was not highlighted as a potential benefit by students who had not revealed their depression.

***Ph.D. students experienced no consequences from revealing their depression to fellow graduate students, and those who concealed only perceived one consequence.*** Notably, none of the 35 students who revealed their depression reported experiencing any consequences associated with revealing their depression to other graduate students, while among the 12 students who concealed their depression there was only one perceived consequence: other graduate students may be reluctant to provide feedback on their research if they know the student has depression (reported by 17%). Students worried others might feel they have to “walk on eggshells” or “sugarcoat their words” when discussing research with a student who has depression.

### Revealing and concealing depression to undergraduate researchers

***Ph.D. students primarily revealed their depression to act as a mentor for undergraduate researchers and to destigmatize mental health.*** Out of 38 students who had interacted with an undergraduate student in their lab, only 14 students (37%) revealed their depression to an undergraduate student and highlighted four common themes why they did so (Table 6**)**. Most graduate student revealing of depression could be categorized as supportive facework because it was meant to help their undergraduates feel respected or secure (Brown, 1977; Ting-Toomey & Cole, 1990; Ting-toomey & Kurogi, 1998). Graduate students most frequently reported that they revealed their depression because they wanted to act as a mentor for undergraduate researchers (50%). Graduate students stated that they felt it was their responsibility to reveal their depression to undergraduates to destigmatize mental health and to show the reality of student experiences while in graduate school. In addition, as was documented with revealing depression to fellow graduate students, participants revealed their depression to undergraduates if they perceived they would not be judged either because they knew the undergraduate also struggled with mental health (43%), or because they had developed a personal relationship with them (36%). Finally, some graduate students engaged in preventative facework, revealing their depression preemptively to undergraduates to avoid conflict or judgement if their depression affected their presence or performance in the lab (14%).

**Table 6:** Reasons why Ph.D. students revealed their depression to undergraduate researchers

Theme	Description	Percent (*N* = 14)^a^	Example student quote 1	Example student quote 2
Supportive reveal	Student wanted to reveal their depression to act as a mentor by normalizing struggling with mental health.	50%	Student 16: “It just felt like my duty to let [the undergraduates] know that graduate school is hard and I'm not going to fake that it's a really happy time so that they know what to expect.”	Student 21: “I think I just wanted [undergraduates] to understand if they were going to choose grad school, the [depressive] feelings they would have, and that they were not alone in them.”
No judgement—other person struggles with mental health	Student reports that they did not think they would be judged for revealing because the undergraduate also struggles with mental health.	43%	Student 4: “I think it was kind of a sense of camaraderie because they were also struggling [with depression]. (…) When somebody tells me, ‘Oh, I'm struggling with depression right now,’ then I can be like, ‘Oh my God, me too.’ And then it feels very supportive.”	Student 15: “One of the undergraduates was like, ‘I have bipolar disorder and I'm really depressed sometimes.’ And I was like, ‘You know what? Thank you for sharing that. Here's the way that I identify with that and here's what we can do together.’”
No judgement—personal relationship	Student reports that they did not think they would be judged for revealing because they had developed a close personal relationship with the undergraduate researcher.	36%	Student 6: "An undergraduate [I mentored in the lab] who worked with me for two years was a friend. I'd known him for a long time, which is why I was able to come clean to him [about my depression]."	Student 19: “I think [the undergraduate and I] have known each other longer than we've worked together so we already had that relationship and that trust built in.”
Preventative reveal	Student perceived that depression would impact or was impacting their mood or performance in research and revealed depression preemptively to avoid judgement or conflict.	14%	Student 29: “I felt bad that I was unprepared. I was late, basically. So, I felt the need to explain why.”	Student 6: “Well, the people I worked over, the undergraduates, needed to know [about my depression] because that was rude of me when I was late.”

^a^Percentages add to more than 100 because students often described more than one reason for revealing their depression to undergraduate researchers

***Ph.D. students primarily concealed their depression from undergraduate researchers because they want to maintain their status as a professional advisor.*** Participants were least likely to reveal their depression to undergraduate researchers, with 63% concealing their depression from them. Graduate students reported that there is a professional barrier between themselves and the undergraduates in their lab, and that as someone who acts as an authority figure, it would be inappropriate to reveal their depression (54%). Graduate students also concealed their depression because they did not feel that they had a close enough relationship with an undergraduate researcher to discuss mental health (38%), and some Ph.D. students perceived their undergraduates were too different from themselves either because of the undergraduate’s age or maturity level (21%). Finally, graduate students worried that by revealing their depression, they could possibly overwhelm the undergraduate researcher and concealed it to avoid unintentionally burdening them (13%). All themes and respective example quotes can be found in Table 7.

**Table 7:** Reasons why Ph.D. students concealed their depression from undergraduate researchers

Theme	Description	Percent (*N* = 24)^a^	Example student quote 1	Example student quote 2
Would break professional barrier	Graduate student describes that they want to maintain their status as a professional, credible, or good advisor, or want to seem more like an authority figure. If they reveal, it could compromise the integrity of their status as an advisor.	54%	Student 48: “I feel an expectation to be in a position of greater wisdom as a superior to [the undergraduates]. So, I think it reflects poorly on my leadership ability to advise them, not in any tangible way, but I think there's an expectation that leaders aren't necessarily supposed to disclose [their depression].”	Student 50: “There is more of a professional barrier between [the undergraduates] than there is between me and other lab mates, so it feels like your boss telling you all this personal information about themselves which might be seen as weird.”
Lack of personal relationship	Graduate student describes that they reveal their depression because they have not established a close enough relationship with the undergraduate.	38%	Student 22 “I have a good relationship with [the undergraduates] and talk to them, but not [a personal] one where I would think about discussing my mental health issues with them.”	Student 24: “Well, [the undergrads and I] are not acquainted as much. I see them sometimes and I train them. So, they're very superficial conversations or conversations about the research and never really about [my depression].”
Perceives cultural/maturity/age difference	Graduate student perceives there is too much of a difference between them and the undergraduate regarding their cultures, ages, or maturity levels to share their depression.	21%	Student 33: “I also am wary of the maturity level where I feel kind of less comfortable talking to [an undergraduate] who might not have the same life experiences as me and might see [my depression] in a different light.”	Student 22: “Well, there is a bit of an age gap between me and most of the undergrads. So, yeah, I don't know if I feel totally comfortable talking to somebody who is 10 years my junior about [my depression].”
Avoid burdening the undergraduate researcher	Graduate student perceives that sharing depression may burden the undergraduate researcher.	13%	Student 46: “I'm pretty sure that [undergraduate researchers] have problems of their own, so they don't need to know and deal with my problems.”	Student 2: “It's always somewhat emotionally taxing to know that somebody is struggling. I would say that pretty much anybody with a standard amount of empathy would feel at least some emotional burden from finding out that somebody that they associate with in some way is dealing with something like depression.”

^a^Percentages add to more than 100 because students often described more than one reason for concealing their depression to undergraduate researchers

***Revealing depression to undergraduate researchers could be beneficial by resulting in increased support or formation of stronger relationships.*** The graduate students who revealed their identity experienced more support from undergraduates in the form of frequent check-ins and increased understanding from the undergraduate researcher (64% of the 14 students who revealed), which was also a benefit that was hypothesized by 46% of the 24 participants who concealed their depression.

***Students who revealed their depression to undergraduates experienced no consequences, while those who concealed their identity perceived that they may be judged by undergraduate researchers if they were to reveal.*** Ph.D. students who revealed their depression to undergraduate researchers reported no consequences, and those who concealed reported the sole potential consequence of experiencing judgment from an undergraduate researcher (17%). Specifically, participants described that an undergraduate researcher may “not take me seriously,” or might “not listen to me as much” if they knew about their depression.

## Discussion

Graduate students were most likely to reveal their depression to fellow graduate students, followed by faculty advisors, and least likely to reveal their depression to undergraduate researchers. Students’ motivation for revealing their depression in the context of graduate research differed based on who they were revealing to.

Graduate students were frequently motivated to share their depression with other graduate students because of the perceived social support and interpersonal relationships, which has consistently been reported as a major influence on an individual’s comfort level with revealing their depression to another individual (Beals et al., 2009; Jones & King, 2014) and is one of the top reasons why people report revealing their CSIs more broadly (Quinn & Earnshaw, 2011). While mutual relationships influenced graduates’ decisions to reveal their depression to their peers, Face Negotiation Theory helps explain their decisions to reveal their depression to both faculty advisors and undergraduates. In both cases, graduate students were navigating hierarchical relationships, meaning that the relationship is based on the individuals’ positions relative to each other. Graduate students primarily revealed their depression to their faculty advisors, those in superior positions, to maintain social dignity. Most commonly, graduate students reported engaging in preventative facework by revealing their depression with the intent to proactively avoid conflict. They also often engaged in corrective facework by revealing their depression to repair their reputation by offering explanations for behavioral changes (Brown, 1977; Ting-Toomey, 1982; Ting-Toomey & Cole, 1990; Ting-toomey & Kurogi, 1998). The decision to preemptively reveal one’s depression aligns with the disclosure process model (Chaudoir & Fisher, 2010). By disclosing one’s depression it likely alleviated psychological and physiological stress because the student no longer had to worry that their advisor would get upset with for lack of productivity or missing meetings (Kranke et al., 2013; White & Labelle, 2019). When engaging with undergraduates, who are in subordinate positions, graduate students primarily engaged in supportive facework by revealing their depression to help undergraduates normalize mental health struggles (Brown, 1977; Ting-Toomey & Cole, 1990; Ting-toomey & Kurogi, 1998), which aligns with reports from undergraduate science students that knowing a scientist with depression can help normalize depression, particularly in the context of science (Cooper et al., 2020a, b). Past literature has found that those in mentorship positions tend to feel responsible to self-disclose parts of their identity to show their willingness to share their own struggles as someone who holds power over another individual (Crisp & Cruz, 2009; Eller et al., 2014). However, graduate students commonly concealed their depression from undergraduate students in effort to maintain social dignity; there were many instances where graduate students were reinforcing a culture that associates depression with weakness (A. H. Crisp et al., 2000; Monteith & Pettit, 2011; Wang & Lai, 2008) and that supports concealing personal information in hierarchical relationships (Brougham & Haar, 2013; Dai et al., 2022; Follmer & Jones, 2018; Peters & Brown, 2009). Together, our data highlight that the impetus for revealing depression changes based on who the graduate student is interfacing with and supports that managing one’s face, or reputation, is often dependent on the nature of the relationship and its hierarchy (Brown, 1977; Ting-Toomey, 1982; Ting-toomey & Kurogi, 1998).

Our study adds to a body of literature highlighting that revealing one’s CSI in a safe environment can result in numerous benefits (2020b; Black & Miles, 2002; Chaudoir & Quinn, 2010; Cooper et al., 2019; Kranke et al., 2013; Quinn & Earnshaw, 2011; Ridge et al., 2019). The graduate students in this study who revealed their depression reported that both faculty advisors and fellow graduate students who knew about their depression provided increased flexibility once they were aware of the Ph.D. student’s depression, often by being more accommodating with deadlines, alleviating responsibilities, and providing additional help on work-related responsibilities. Notably, when we asked students who concealed their depression about possible benefits, only 20% identified this as a possible benefit of revealing to a faculty advisor and no student identified it as a possible benefit for revealing to graduate students. Relatedly, Ph.D. students who revealed their depression highlighted that they were able to be more honest with their faculty advisor and fellow graduate students in situations where their depression limited their ability to complete research tasks. However, this was also never mentioned by Ph.D. students who concealed their depression as a potential benefit. Therefore, these may be unexpected benefits of revealing depression that Ph.D. students may not consider as they weigh the decision to conceal or reveal. In addition to the positive impact that revealing depression appeared to have on graduate students themselves, there is reason to believe that the act of revealing one’s CSI may have also benefitted others, particularly undergraduates. A study of 35 undergraduate researchers with depression revealed that only one-third of students knew a scientist with depression (Cooper et al., 2020b). However, undergraduates who did not know a scientist with depression reported that if they did, it would serve as proof that individuals with depression can be successful in science in addition to helping them feel less alone as someone experiencing depression in the context of research. Recent research suggests that when an instructor reveals a CSI in academic science, students who share that CSI may benefit disproportionately (Busch et al., 2022b) and this has been shown to be particularly true for students with depression (Mohammed et al., unpublished data). In a study of 289 biology undergraduates, an instructor briefly revealed their depression in an upper-level physiology course (Mohammed, unpublished data). Researchers found that compared to biology undergraduates without depression, biology undergraduates with depression disproportionately reported that they were positively impacted by the instructor revealing their depression and were more likely to report that the instructor coming out had a positive effect on normalizing depression broadly and normalizing depression specifically in the context of science. Similar sentiments may be shared among undergraduate researchers who come to know graduate researchers with depression. In sum, the graduate students revealing their depression to others in the context of research has the potential to yield benefits that extend beyond themselves.

Consequences of revealing depression were occasionally reported by students who revealed their identity. The consequences that students who concealed their depression anticipated aligned closely with their reasons for concealing their identities, including concerns about being judged, being unfairly relieved of responsibilities, and being treated differently, which each echo concerns that undergraduates researchers have voiced about sharing their depression in research labs (Cooper et al., 2020b). Participants most commonly reported that they concealed their depression from both their faculty advisor and fellow graduate students because they perceived they may be viewed or treated negatively, likely owing to the stigma that people with depression are weak and unpredictable (A. H. Crisp et al., 2000; Monteith & Pettit, 2011; Wang & Lai, 2008). Students who concealed their depression because they anticipated being stripped of responsibilities if they were to reveal demonstrated identity interference where they perceive that their depression interferes with their identity as a scientist (National Academies of Sciences, Engineering, & Medicine, 2019). Ph.D. students also commonly reported concealing their depression because they were personally uncomfortable with it. One reason for such discomfort is internal stigma, which is often a result of learning stereotypes about depression early in one’s life (Link, 1987; Quinn & Earnshaw, 2011; Ritsher & Phelan, 2004) because depression is often depicted in television shows as unattractive, violent, and criminal (Wahl, 2003) and sometimes referenced by family and friends in a derogatory way (Dovidio, 2010). Some Ph.D. students perceived revealing their depression in the context of research to be unnecessary, which aligns with literature highlighting that if one perceives their CSI will not interfere with their work, it is irrelevant to reveal in that particular context (Kranke et al., 2013; Meluch & Starcher, 2020). In addition, although a number of Ph.D. students viewed revealing depression as an important part of mentoring, others felt the need to conceal it to establish a professional barrier. Indeed, prior literature has highlighted that individuals who serve in advisor roles often perceive that it is necessary to conceal stigmatized identities to keep professionalism intact (Anderson & Shore, 2008; Ridge et al., 2019). This may be further exacerbated in the generally apathetic culture in both science and academia that encourages separation of work and emotion (Christopher Strenta et al., 1994; Ebenezer & Zoller, 1993). Notably, graduate students rarely reported that they revealed their depression because other undergraduates or faculty mentors revealed their mental health struggles first. We hypothesize that this is likely because of how infrequently undergraduates and faculty choose to reveal their depression (Cooper et al., 2020b, Busch et al., 2023). Further research needs to be done to understand how frequently undergraduates and faculty reveal their depression to graduate students specifically in order to better understand the magnitude of that decision on graduate students’ decisions to reveal their own depression.

### Implications

This research suggests that creating spaces where science graduate students feel safe revealing their depression may benefit graduate students in a variety of ways including allowing them to be more transparent about their struggles and bolstering emotional support from lab mates and mentors. Furthermore, more graduate students revealing their depression may help to normalize mental health struggles among undergraduate science students (Cooper et al., 2020b), which may disproportionately impact students who are underrepresented and underserved in science because of their likelihood for also having depression (Busch et al., 2022a). Our research reveals that one of the most important steps advisors can take to make graduate students feel comfortable revealing their depression is to indicate an understanding of mental health. For example, openly acknowledging that graduate students should take care of their mental health as they do their physical health may help establish that a mentor values students’ mental health (Cooper et al., 2020a; Gin et al., 2021). There is increasing evidence that faculty revealing their own CSIs, such as depression, can positively impact students, particularly those who share the CSI (Busch et al., 2022b; Cooper et al., 2019; Cooper & Brownell, 2016; Mohammed et al., unpublished data). While it was difficult to discern the impact of faculty revealing depression to graduate students in this study, likely owing to the paucity of graduate mentors who talk about their depression, mentors may want to consider the positive implications of being open about their own mental health struggles. In addition, the extent to which graduate students feel as though they establish relationships with their mentors, peers, and undergraduate mentees influenced the extent to which they reveal their depression. As such, efforts at the institutional, department, and lab level to create community among individuals in the sciences may be particularly helpful in making students feel more comfortable talking about mental health. Because depression disproportionately affects individuals who are underrepresented and underserved in science (American College Health Association, 2018; Eisenberg et al., 2007; Evans et al., 2018; Jenkins et al., 2013; Turner & Noh, 1988), this study highlights that making efforts to create a community where graduate students can feel comfortable with and benefit from revealing their depression, may ultimately help lead to a more diverse community of scientists, resulting in more robust science (Intemann, 2009). Importantly, choosing to reveal one’s depression is a deeply personal decision and may result in consequences; however, we hope that this work will aid graduate students in making the decision about revealing their depression that is right for them.

## Limitations

While this study explored the benefits and consequences associated with revealing one’s depression, we did not explore the benefits or challenges associated with concealing one’s depression. Owing to the study design, we were unable to assess whether students are unaware of potential benefits of revealing depression, which lessens the chance that they will reveal, or whether students who are less likely to benefit are also less likely to reveal. In addition, it was beyond the scope of this study to investigate the correlations between student demographics and their experiences with revealing or concealing their depression owing to the small number of students in different demographic groups. However, such relationships should be investigated in future large-scale quantitative studies.

## Conclusion

We conducted interviews with 50 life sciences Ph.D. students to examine to what extent and why they chose to reveal or conceal their depression to faculty, fellow graduate students, and undergraduate researchers. Nearly three-quarters of Ph.D. students reported having revealed their depression to another graduate student, and over half revealed their depression to their faculty advisor. Graduate students were least likely to report revealing their depression to undergraduate researchers within their lab. Students’ decisions to reveal their depression to their peers were driven by strong mutual relationships, while their decision to reveal their depression to faculty advisors was driven by preventative or restorative facework, and their decision to reveal to undergraduate researchers was driven by supportive facework. This research highlights the benefits of being able to work in a research environment where one feels comfortable revealing their depression and emphasizes the impact of hierarchical relationships on graduate students’ decisions to reveal or conceal their depression in research.

## Supplementary Information

**Additional file 1. **Copy of interview script. Additional tables.

## Data Availability

Underlying data are subject to ethical restrictions as the interview transcripts contain identifiable information, including student names, the names of PIs, graduate, and undergraduate researchers, and anecdotes that could identify the students. Furthermore, the interviews include sensitive information about students’ mental health; students were assured before their interview that their transcripts would never be shared with anyone outside of the research team. It is for these reasons that the data will not be shared. However, the interview protocol is available in the Additional file 1.
