# Supplementary material for: Face negotiation in graduate school: the decision to conceal or reveal depression among life sciences Ph.D. students in the United States
Source: Int J STEM Educ. 2023 May 17;10(1):35. doi: 10.1186/s40594-023-00426-7 (PMC10189696; doi:10.1186/s40594-023-00426-7)
Supplement: Supplementary file 1 — Additional file 1. Copy of interview script. Additional tables. [file 40594_2023_426_MOESM1_ESM.pdf]

Additional information for:

Face negotiation in research: The decision to conceal or reveal depression among life sciences Ph.D. students

This supplement contains the following:

Item	Page
Copy of final interview script	2
Copy of survey questions	4
Additional file 1: Table S1 and query results	7
Additional file 1: Table S1-10. Copy of final coding rubrics showing occurrences of themes	10

Copy of interview script

To what extent do you interact with your faculty research advisor/PI?

Does your **faculty** research advisor know about your depression?

- If yes:
  - How did they find out?
  - What made you feel comfortable revealing your depression to those individuals?
  - Were there any instances where you felt that they treated you differently in a **negative** way after they knew about your depression?
  - Were there any instances where you felt that they treated you differently in a **positive** way after they knew about your depression?

- If no:
  - Why not?
  - What reasons, if any, made you feel uncomfortable revealing your depression to those individuals?
  - If they did know about your depression, do you think there would be instances where they would treat you differently in a **negative** way because of your depression?
  - If they did know about your depression, do you think there would be instances where they would treat you differently in a **positive** way because of your depression?

Do you have other grad students or post-docs in your Ph.D. research group that you interact with?

Do any **grad students or postdocs in your research group** know about your depression?

- If yes:
  - How many people know?
  - How did they find out?
  - What made you feel comfortable revealing your depression to those individuals?

    - Were there any instances where you felt that they treated you differently in a **negative** way after they knew about your depression?
    - Were there any instances where you felt that they treated you differently in a **positive** way after they knew about your depression?
- If no:
    - Why not?
    - Were there any reasons that you felt uncomfortable revealing your depression to those individuals?
    - If they did know about your depression, do you think there would be instances where they would treat you differently in a **negative** way because of your depression?
    - If they did know about your depression, do you think there would be instances where they would treat you differently in a **positive** way because of your depression?

Do you have any undergraduates in your Ph.D. research group that you interact with?

Do any of the **undergraduate researchers in your Ph.D. research group know** about your depression?

- If yes:
    - How many?
    - How did they find out?
    - What made you feel comfortable revealing your depression to those individuals?
    - Were there any instances where you felt that they treated you differently in a **negative** way after they knew about your depression?
    - Were there any instances where you felt that they treated you differently in a **positive** way after they knew about your depression?

- If no:
    - Why not?
    - Were there any reasons that you felt uncomfortable revealing your depression to those individuals?
    - If they did know about your depression, do you think there would be instances where they would treat you differently in a **negative** way because of your depression?

If they did know about your depression, do you think there would be instances where they would treat you differently in a **positive** way because of your depression?

Is there anything else you would like to share?

Copy of student-level demographic survey questions
I most closely identify as

- Woman
- Man
- Non-binary/Gender fluid
- Other (please describe)
- Decline to state

I most closely identify as

- American Indian or Alaska Native
- Asian
- Black or African American
- Hispanic, Latinx, or Spanish origin
- Pacific Islander
- White/Caucasian
- Other (please describe)
- Decline to state

What is your parent's highest completed level of education? If you have more than one parent with differing levels of education, choose the higher of the two.

- Did not complete high school
- High school diploma or GED
- Some college but no degree
- Associate degree (for example: AA, AS)
- Bachelor's degree (for example: BA, AB, BS)
- Master's degree (for example: MA, MS, MEng, MEd, MSW, MBA)
- Higher than a Master's degree (for example: PhD, MD, JD)
- Other (please describe)
- Decline to state

Are you an international student?

- Yes (If yes, which country are you from?)
- No
- Decline to state

How old are you?

What is your career goal? (Please list)

Copy of research/teaching-level demographic survey questions
How long have you been in your PhD program?
- Less than one year
- One year
- Two years
- Three years
- Four years
- Five years
- Six years
- More than six years
- I have finished my PhD program

What is the focus area of your research? (e.g., animal behavior, neurobiology, cancer bio)

Do you any have experience teaching undergraduate students? For example, as a Teaching Assistant.
- Yes (If yes, how many courses have you taught either as an instructor of record or as a teaching assistant?)
- No

Copy of depression demographic survey questions

For these interviews, we are interested in the experiences of **all students, regardless of whether they have been formally diagnosed with depression,** and **regardless of whether they are being treated with depression**. Thus, there are no right or wrong answers to these questions. We are simply trying to learn more about your experience.

During your graduate research experience, in your opinion how severe is/was your depression?

- Mild
- Moderate
- Severe
- Extremely severe
- Decline to state

Have you been diagnosed with depression?

- Yes
- No
- Decline to state

Are you currently being treated for, or have you previously been treated for depression?

- Yes
- No
- Decline to state

Additioanl file 1: Table S1 and query results

Additional file 1: Table S1. Each interview participant, whether they have been formally diagnosed with depression, the severity of their depression during graduate school, their year in program, and whether they had revealed their depression to the faculty mentor, at least one graduate students, and to at least one undergraduate.

Student #	Diagnosed with depression	Severity of depression	Year in program	Reveal to faculty	Reveal to graduate students/postdocs[a]	Reveal to undergrads[a]
Student 1	No	Mild	4 years	−	✓	✓
Student 2	Yes	Moderate	3 years	−	✓	−
Student 3	Yes	Moderate	4 years	✓	✓	−
Student 4	Yes	Moderate	2 years	✓	✓	✓
Student 5	Yes	Severe	Finished PhD	✓	✓	✓
Student 6	No	Severe	Finished PhD	✓	✓	✓
Student 7	Yes	Moderate	5 years	✓	✓	−
Student 8	Yes	Moderate	2 years	✓	✓	−
Student 9	Yes	Moderate	2 years	✓	✓	N/A
Student 10	Decline to state	Decline to state	2 years	✓	✓	−
Student 11	No	Severe	<1 year	−	✓	−
Student 12	Yes	Moderate	6+ years	✓	✓	✓
Student 13	No	Severe	4 years	✓	−	N/A
Student 14	Yes	Moderate	1 year	−	✓	N/A
Student 15	Yes	Severe	2 years	✓	✓	✓
Student 16	Yes	Extremely severe	2 years	✓	✓	✓
Student 17	No	Mild	2 years	−	✓	N/A
Student 18	Yes	Mild	4 years	−	−	−

Student 19	Yes	Severe	2 years	✓	−	✓
Student 20	Yes	Moderate	3 years	✓	−	−
Student 21	Yes	Moderate	Finished PhD	−	✓	✓
Student 22	No	Mild	3 years	−	−	−
Student 23	Yes	Severe	6+ years	✓	✓	✓
Student 24	Yes	Severe	1 year	−	✓	−
Student 25	Yes	Extremely severe	5 years	✓	✓	✓
Student 26	Yes	Severe	6+ years	✓	✓	−
Student 27	Yes	Moderate	4 years	−	N/A	−
Student 28	No	Moderate	2 years	−	N/A	N/A
Student 29	Yes	Moderate	5 years	✓	✓	✓
Student 30	Yes	Severe	6+ years	−	−	−
Student 31	Yes	Moderate	3 years	✓	✓	−
Student 32	Yes	Moderate	3 years	−	✓	✓
Student 33	Yes	Moderate	3 years	✓	✓	−
Student 34	No	Moderate	3 years	✓	✓	N/A
Student 35	Yes	Severe	2 years	✓	✓	−
Student 36	Yes	Extremely severe	3 years	✓	N/A	−
Student 37	Yes	Severe	1 year	✓	−	N/A
Student 38	Yes	Moderate	2 years	✓	✓	N/A
Student 39	Yes	Mild	3 years	−	✓	N/A
Student 40	Yes	Severe	6+ years	✓	✓	−
Student 41	Decline to state	Decline to state	2 years	−	−	N/A
Student 42	Yes	Mild	5 years	✓	✓	✓
Student 43	Yes	Moderate	5 years	−	✓	−
Student 44	Yes	Moderate	3 years	−	−	N/A
Student 45	Yes	Severe	2 years	−	−	N/A
Student 46	Yes	Moderate	5 years	✓	✓	−

Student 47	Yes	Moderate	6+ years	−	−	−
Student 48	Yes	Moderate	5 years	−	✓	−
Student 49	Yes	Moderate	3 years	✓	✓	−
Student 50	Yes	Mild	3 years	−	−	−
[a]N/A: indicates that a student reported having never worked with this type of individual and thus did not have the opportunity to reveal their depression						

**Query 1: Depression severity**	
**Group**	**% reveal**
Mild	32
Moderate	58
Severe	62
Extremely severe	91

**Query 2: Year in program**	
**Group**	**% reveal**
1	40
2	69
3	44
4	38
5	71
6+	42
Finished PhD	88

**Query 3: Formal diagnosis**	
**Group**	**% reveal**
Yes	59
No	59

Grad/faculty relationship

Reasons why Ph.D. students revealed their depression to their faculty advisor.

Theme	% of students who reported the theme (N = 29)	Description
Preventative reveal	48	Student perceived that depression would impact or was impacting their mood or performance in research and revealed depression preemptively to avoid judgement or conflict. This includes if a student thinks other people might suspect something may be wrong and so they want to get ahead of it by revealing their depression before someone approaches them about it.
Corrective reveal	41	Student reports that depression negatively affected their mood or performance in research, which was noticed by others. The student revealed their depression in an attempt to explain their mood or behavior after it was noticed or commented on by others. The major difference between a preventative reveal and a corrective reveal is that corrective reveals only happen once another person has mentioned a problem or concern.
No judgment - understanding	22	Student perceived that they would not be judged if they revealed because their advisor's past actions indicated that the advisor is understanding, caring, or has a positive perception of mental health.
No judgment - personal	17	Student perceived that they would not be judged for revealing because they had developed a close personal relationship with their faculty advisor.

relationship		
No judgement: Other person struggles with mental health	7	Student knows or perceives that the person they are sharing their depression with also has mental health issues- often depression but may be another mental health concern such as anxiety. Students often describe that shared mental health experiences decrease the chance they will be judged negatively for their depression.

Reasons why Ph.D. students concealed their depression from their faculty advisor.

Theme	% of students who reported the theme (N = 21)	Description
May be perceived or treated differently or in a negative way	57	Student describes they were afraid they would be perceived or treated negatively if their advisor knew of their depression. Students often report using their advisor's prior behaviors to predict how they would react. This includes a fear of being treated negatively (e.g. being judged) or differently (having people "walk on eggshells" around them).
Is uncomfortable with depression	38	Student describes that they are personally uncomfortable sharing their depression with anyone, so they would not share it with their faculty advisor.
Perceives revealing to be unnecessary	29	Student did not think it was necessary to share their depression, often because they did not perceive it affected their work.

Perceives an identity cultural or age difference between them and their advisor	19	Student does not reveal because they worry that the faculty advisor's identity or culture would prevent them from understanding what the student is trying to explain. This includes faculty who had an easy time in graduate school or expressed that they remember graduate school being extremely fun and exciting, because these experiences do not align with those of the graduate student.
Perceives revealing to be inappropriate	14	Student perceives that it is inappropriate to reveal depression in a lab environment, often because of the assumption that emotion is not welcome in science.
Not enough of a personal relationship	9	Student describes that they don't talk about their depression because they haven't established a close enough relationship with another person they could reveal to.
Student worries that their depression might be revealed to others	9	Student is worried that the person in question may reveal their depression to others that they do not want to reveal to.
Doesn't want to be a burden to faculty	9	Student describes that they don't want faculty mentor to worry about the student or make adjustments to the student's work.

Ph.D. student perceived benefits from revealing their depression to their faculty mentor

Benefits	% of students who	% of students who	Description

	revealed who reported the theme (N = 29)	concealed who reported the theme (N = 21)	
More flexible	48	19	Makes a change to accommodate student depression (e.g., extends deadline, modifies responsibilities). Student describes that person is more understanding of student's depression, actions or behaviors (e.g., missing deadlines, missed meeting, not accomplishing goals/tasks).
More support	48	43	Person provides additional mentorship/guidance (in research or personally), checks in on student, or can even provide mental health resources
More honest	10	0	Student is able to be honest about when they cannot fulfill an obligation, come into the lab, make a meeting, etc...

Ph.D. student consequences and perceived risks of revealing their depression to their faculty mentor

Risks	% of students who revealed who reported the theme (N = 29)	% of students who concealed who reported the theme (N = 21)	Description
Judged	21	24	Student reports that they felt judged by the faculty mentor and the faculty mentor has a negative view of them after

			revealing their depression. This is distinct from other categories which involve the person acting on those judgments.
Relieves/ Prevents student of responsibilities	0	14	Faculty relieves student of responsibilities, doesn't give student the standard work they want, doesn't put student forward for opportunities

Grad student/peer relationship

Reasons why Ph.D. students revealed their depression to other graduate students.

Theme	% of students who reported the theme (N = 35)	Description
No judgement - personal relationship	49	Student reports that they did not think they would be judged for revealing because they had developed a close personal relationship with a fellow graduate student.
No judgement - other person struggles with mental health	43	Student reports that they did not think they would be judged for revealing because the other graduate student also struggles with mental health.
No judgement – shared (often negative) experiences in graduate school	20	Student reports that they did not think they would be judged for revealing because they shared similar experiences, often negative ones, during graduate school.
Supportive reveal	20	Student wanted to reveal their depression to normalize struggling with mental health.
Preventative reveal	17	Student perceived that depression would impact or was impacting their mood or performance in research and revealed depression preemptively to avoid judgement or conflict.
Corrective	11	Student reports that depression negatively affected their mood or

reveal		performance in research, which was noticed by others. The student revealed their depression in an attempt to explain their mood or behavior after it was noticed or commented on by others.
No judgement: Person is understanding/caring/positive outlook on MH	9	Student perceives that they will likely not be judged because the person's past actions have indicated that they are understanding/caring/have a positive outlook on mental health.

Reasons why Ph.D. students concealed their depression from other graduate students.

Theme	% of students who reported the theme (N = 12)	Description
Lack of personal relationship	58	Student describes that they did not reveal their depression because they did not establish a close enough relationship with other graduate students.
May be perceived or treated in a negative way	33	Student describes they were afraid they would be perceived or treated negatively if other graduate students knew of their depression. Students often report using their fellow graduate students' prior behaviors to predict how they would react.

Perceives revealing to be unnecessary	17	Student did not think it is necessary to share depression, often because they did not perceive it affected their work.
Is uncomfortable with depression	17	Student describes that they are personally uncomfortable sharing their depression with anyone.
Student worries that their depression might be revealed to others	8	Student is worried that the person in question may reveal their depression to others that they do not want to reveal to.
Misalignment of identity or culture between the student revealing and the person they're revealing to	8	Student does not reveal because they worry that the other graduate student's identity or culture prevents them from understanding what they are trying to say.
Doesn't want to be a burden to graduate student	0	Student describes that they don't want other graduate student to have to worry about the student or make adjustments.
Inappropriate: Research is a professional setting (no place for emotion)	0	Student feels that the lab/academia is no place for emotion, that it is inappropriate to share depression, etc.

Ph.D. student-perceived benefits of revealing their depression to other graduate students.

Benefits	% of	% of	Description

	students who revealed who reported the theme (N = 35)	students who concealed who reported the theme (N = 12)	
More support	80	67	Person provides additional community, stronger friendship, opportunities to commiserate or to feel included. This also includes checking in with a student or sharing mutual experiences or hardships.
More honest	14	0	Student is able to be honest about when they cannot fulfill an obligation, come into the lab, make a meeting, etc.

Ph.D. student-perceived consequences of revealing their depression to other graduate students

Benefits	% of students who revealed who reported the theme (N = 35)	% of students who concealed who reported the theme (N = 12)	Description
Reluctant to provide feedback	0	17	Student describes that fellow graduate students may be reluctant to be honest with them about their research if they were to know about their depression. Students also describe

			worrying that people will not share criticisms and critiques of their work in fear they will exacerbate their depression.

Grad student/undergrad relationship

Reasons why Ph.D. students revealed their depression to undergraduate researchers.

Theme	% of students who reported the theme (N = 14)	Description
Supportive reveal	50	Student wanted to reveal their depression to normalize struggling with mental health.
No judgement - other person struggles with mental health	43	Student reports that they did not think they would be judged for revealing because the undergraduate also struggles with mental health.
No judgement - personal relationship	36	Student reports that they did not think they would be judged for revealing because they had developed a close personal relationship with the undergraduate researcher.
Preventative reveal	14	Student perceived that depression would impact or was impacting their mood or performance in research and revealed depression preemptively to avoid judgement or conflict.
Corrective reveal	7	Student reports that depression negatively affected their mood or performance in research, which was noticed by others. The student revealed their depression in an attempt to explain their mood or behavior after it was noticed or commented on by others.
No judgement – shared (often	7	Student reports that they did not think they would be judged for revealing because they shared similar experiences, often negative ones,

negative) experiences in graduate school		during graduate school and as an undergraduate.
No judgement - other person struggles with mental health	7	Student reports that they did not think they would be judged for revealing because the undergraduate researcher also struggles with mental health.

Reasons why Ph.D. students concealed their depression from undergraduate researchers.

Theme	% of students who reported the theme (N = 24)	Description
Would break professional barrier	54	Student describes that they want to maintain their status as a professional, credible, or good advisor or want to seem more like an authority figure. If they reveal, it could compromise the integrity of their status as a advisor.
Lack of personal relationship	38	Student describes that they reveal their depression because they have not established a close enough relationship with the undergraduate.
Perceives cultural/	21	Student perceives there is too much of a difference between them and

maturity/age difference		the undergraduate regarding the culture between the undergrad and grad, their ages, or their maturity levels to share their depression.
Avoid burdening the undergraduate researcher	13	Student perceives that sharing depression may burden the undergraduate researcher.
May be perceived or treated in a negative way	8	Student describes they were afraid they would be perceived or treated negatively if undergraduate researchers knew of their depression. Students often report using the undergraduate researcher's prior behaviors to predict how they would react.
Is uncomfortable with depression	4	Student describes that they are personally uncomfortable sharing their depression with anyone.

Ph.D. student-perceived benefits in revealing their depression to undergraduate researchers.

Benefits	% of students who revealed who reported the theme (N = 14)	% of students who concealed who reported the theme (N = 24)	Description
More support	64	46	Person provides additional community, stronger friendship, opportunities to commiserate or to feel included. This also includes checking in with a student or sharing mutual experiences or hardships.

Ph.D. student-perceived consequences of revealing their depression to other graduate students

Benefits	% of students who revealed who reported the theme (N = 14)	% of students who concealed who reported the theme (N = 24)	Description
Judgement	0	17	Student describes that undergraduates may judge them negatively for having depression.
